# Perinatal grief caracteristics

**DOI:** 10.1192/j.eurpsy.2021.1609

**Published:** 2021-08-13

**Authors:** R. Pinilla, R. Hermosillo

**Affiliations:** Psiquiatria, Hospital de Getafe, Madrid, Spain

**Keywords:** Grief, perinatal, pregnance

## Abstract

**Introduction:**

Perinatal grief is the reaction to the death of a loved one in the perinatal period (according to the WHO, it ranges from 22 weeks of gestation to the 1st week of postnatal life). Despite the fact that perinatal grief presents a set of distinctive characteristics, it is not recognized as a differentiated entity in the main diagnostic manuals (DSM-5 and ICD-11). There are a number of characteristics that make perinatal grief a different grief reaction. Characteristics that make perinatal grief a different grief reaction:

General characteristics: Proximity between the beginning and the end of life, the lack of religious rituals that legitimize the loss. Physiopathological characteristics; The gestational hormone increase act in the brain favoring emotional bonding with the child and facilitating care, sustained modifications in the gabaergic, endorphinic and nitrinergic synapses in the mothers’ brains. Increased physical activity of the fetus during the third trimester increases the mother’s basal metabolism and changes her emotional reaction. Clinical characteristics; feelings of guilt, loneliness and detachment, irritability, dissociative symptoms, concern dead son and angry reactions.

**Objectives:**

Search for the specific characteristics of perinatal grief and the importance of its therapeutic approach.

**Methods:**

Literature review using pubmed database and scientific dissemination articles.

**Results:**

Between 10 and 50% of mothers who suffer perinatal grief develop depression disorder, 50% have anxiety disorders that usually reappear with the possibility of a new pregnancy, and between 5 and 25% are diagnosed with post-traumatic stress disorder.

**Conclusions:**

Perinatal grief has characteristics that differentiate it from other grief reactions; mental health professionals must attend to and understand these specificities in order to attend it.

